# Amblyopia treatment of adults with dichoptic training using the virtual reality oculus rift head mounted display: preliminary results

**DOI:** 10.1186/s12886-017-0501-8

**Published:** 2017-06-28

**Authors:** Peter Žiak, Anders Holm, Juraj Halička, Peter Mojžiš, David P Piñero

**Affiliations:** 1 Eye clinic, Jessenius faculty of Medicine in Martin, Commenius University in Bratislava, Bratislava, Slovakia; 2 UVEA Mediklinik, Martin, Slovakia; 3 Premium Clinic, Teplice, Czech Republic; 4 Department of Optics, Pharmacology and Anatomy, University of Alicante, Alicante, Spain

**Keywords:** Amblyopia, Dichoptic training, Virtual reality, Stereopsis, Oculus rift

## Abstract

**Background:**

The gold standard treatments in amblyopia are penalizing therapies, such as patching or blurring vision with atropine that are aimed at forcing the use of the amblyopic eye. However, in the last years, new therapies are being developed and validated, such as dichoptic visual training, aimed at stimulating the amblyopic eye and eliminating the interocular supression.

**Purpose:**

To evaluate the effect of dichoptic visual training using a virtual reality head mounted display in a sample of anisometropic amblyopic adults and to evaluate the potential usefulness of this option of treatment.

**Methods:**

A total of 17 subjects (10 men, 7 women) with a mean age of 31.2 years (range, 17–69 year) and anisometropic amblyopia were enrolled. Best corrected visual acuity (BCVA) and stereoacuity (Stereo Randot graded circle test) changes were evaluated after 8 sessions (40 min per session) of dichoptic training with the computer game Diplopia Game (Vivid Vision) run in the Oculus Rift OC DK2 virtual reality head mounted display (Oculus VR).

**Results:**

Mean BCVA in amblyopic eye improved significantly from a logMAR value of 0.58 ± 0.35 before training to a post-training value of 0.43 ± 0.38 (*p* < 0.01). Forty-seven percent of the participants achieved BCVA of 20/40 or better after the training as compared to 30% before the training. Mean stereoacuity changed from a value of 263.3 ± 135.1 before dichoptic training to a value of 176.7 ± 152.4 s of arc after training (*p* < 0.01). A total of 8 patients (47.1%) before dichoptic treatment had unmeasurable stereoacuity while this only occurred in 2 patients (11.8%) after training.

**Conclusions:**

Dichoptic training using a virtual reality head mounted display seems to be an effective option of treatment in adults with anisometropic amblyopia. Future clinical trials are needed to confirm this preliminary evidence.

**Trial registration:**

Trial ID: ISRCTN62086471. Date registered: 13/06/2017. Retrospectively registered

## Background

Amblyopia is a reduction of the best corrected visual acuity of the eye without organic cause [1]. In this condition, there is an abnormal binocular experience due to a mismatch between the images perceived with each eye [1]. This situation may be caused by visual deprivation due to congenital cataract, unequal refractive errors or strabismus [1].The physiology of the retina is generally spared in amblyopia, with visual pathway changes linked to the geniculate and post-geniculate part [2]. Mean prevalence of amblyopia is estimated to be 2–5% [3]. Permanent monocular visual impairment due to amblyopia is a risk factor for blindness if the dominant eye is injured or if the fellow eye is affected by disease later in life [4]. For this reason, the early treatment of this condition is crucial. The gold standard treatments in amblyopia are penalizing therapies, such as patching or blurring vision with atropine that are aimed at forcing the use of the amblyopic eye [5]. This type of amblyopia treatment can be effective for up to 7 years of age [6].

Recovery of normal visual functions is thought to be almost impossible after critical period ends, i.e., after 8 years of age in children. However, there exist several animal and human studies that show visual pathway plasticity even after critical period has passed, being patients who have lost vision in the “good” eye some examples of this [7–9]. The loss of the fellow eye would allow the existing connections to be reactivated. This could be the result of unmasking [10] or higher brain areas learning to attend to the previously inhibited signals from the amblyopic eye. Vision therapy after the end of the critical period may result in improvement in vision and binocularity. Studies on video games played by adults for the treatment of amblyopia have shown some degree of visual restoration in the amblyopic eye [11, 12]. Thus, the critical period should be only considered as the time of maximum neurological plasticity. We have conducted a preliminary study evaluating the effect of dichoptic visual training using a virtual reality head mounted display in a sample of amblyopic adults in order to evaluate the potential usefulness of this option of treatment.

## Methods

A total of 17 amblyopic subjects (10 men, 7 women) with a mean age of 31.2 years (range, 17–69 year) were enrolled in this study. Inclusion criteria were subjects with anisometropic amblyopia, age of 17 years old or more and willing to perform the visual training. Exclusion criteria strabismus, previous ocular surgery, corneal irregularity, opacification of ocular media including cataracts and active ocular disease. All patients were informed about the study and provide a written informed consent following the tenets of the Declaration of Helsinki. The study protocol was approved by the Ethics Committe of Jessenius Medical School, Commenius University in Martin.

All patients underwent a baseline ophthalmological examination including visual testing, manifest and cycloplegic refraction, cover test, four dot Worth test, anterior segment examination with the slit lamp, corneal topography, and funduscopy. Best corrected visual acuity (BCVA) was measured using a calibrated liquid crystal display (LCD) optotype with Snellen charts (CC-X10, Topcon, Japan). The stereoacuity was measured using the Stereo Randot graded circle test (Stereo Optical, IL, USA). BCVA and stereoacuity were measured before and after the program of dichoptic training.

**Fig. 1:**
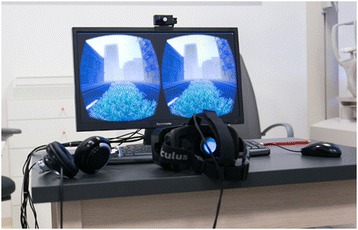
Oculus Rift DK2 setup. On the LCD screen what patient sees inside head mounted display is shown

**Fig. 2:**
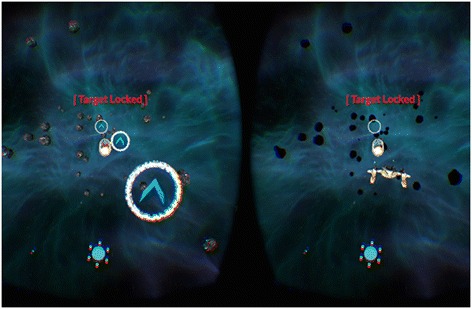
Example of the dichoptic training game seen through the oculus rift head mounted display in virtual reality. The amblyopic eye views the left half of the display in which the patient sees the correct color of the gates in order to flight spaceship throught the blue gates. Spaceship is seen only with the dominant eye, the right part of the figure

Dichoptic visual training was performed using the beta version of the computer game Diplopia Game (Vivid Vision, San Francisco, USA) which was run in the Oculus Rift OC DK2 virtual reality head mounted display (Oculus VR, LLC, Irvine, California, USA). The OC DK2 was equiped with an AMOLED display (5,7″ diagonal, resolution of 960 × 1080 pixels per eye), with 100° field of view, mounted with accelerometer, gyroscope and magnetometer sensor for positional tracking system (Fig. 1). Virtual reality head mounted display Oculus Rift was connected to a PC system (Intel i5 3,4 GHz, 8 GB RAM, Nvidia GeForce 970GT 4GB). Two games were available, a space game in which subjects were flying spaceship through a system of rings and a breaker game which is a typical block breaker game, but played in a virtual reality 3D setting. Both games had a dichoptic setting in which the central part of the picture shown was different. In the space game, spaceship was visible only with the fixating eye whereas colorful gates and asteroids were only visible with the amblyopic eye (Fig. 2). The spaceship is only seen with the dominant eye to avoid cheating. If all objects of the game are seen with the amblyopic eye, the patient can just close the dominant eye and “cheat”. As some objects are seen with the amblyopic eye and others are seen with the fellow eye, the game forces the brain to use both eyes together to play.

Each subject underwent 8 training sessions, being performed twice a week. Each session included 40 min of training with 2 different games (20 min per game). Optometric tests that were available in the beta version of the software were performed directly in the head mounted display before each training (ocular dominance and suppression). BCVA was tested before first and after last training session. Patient did not perform any other visual training during the period of dichoptic training. Ten patients were treated with patching when they were child, but they did not remember for how long. Data analysis was performed using the software SPSS for Windows version 19.0 (IBM, Armonk, NY, USA). The status of normality of the data was determined using the Kolmogorov-Smirnov test. When the assumptions of normality were met, the Student’s t-test was used for paired samples to check the differences between before and after training visits. Otherwise, when normality assumptions were not met, the Wilcoxon signed-rank test was used to analyse the differences between follow-up visits. A *p*-value of less than 0.05 was considered as statistically significant for all tests.

## Results

**Fig. 3:**
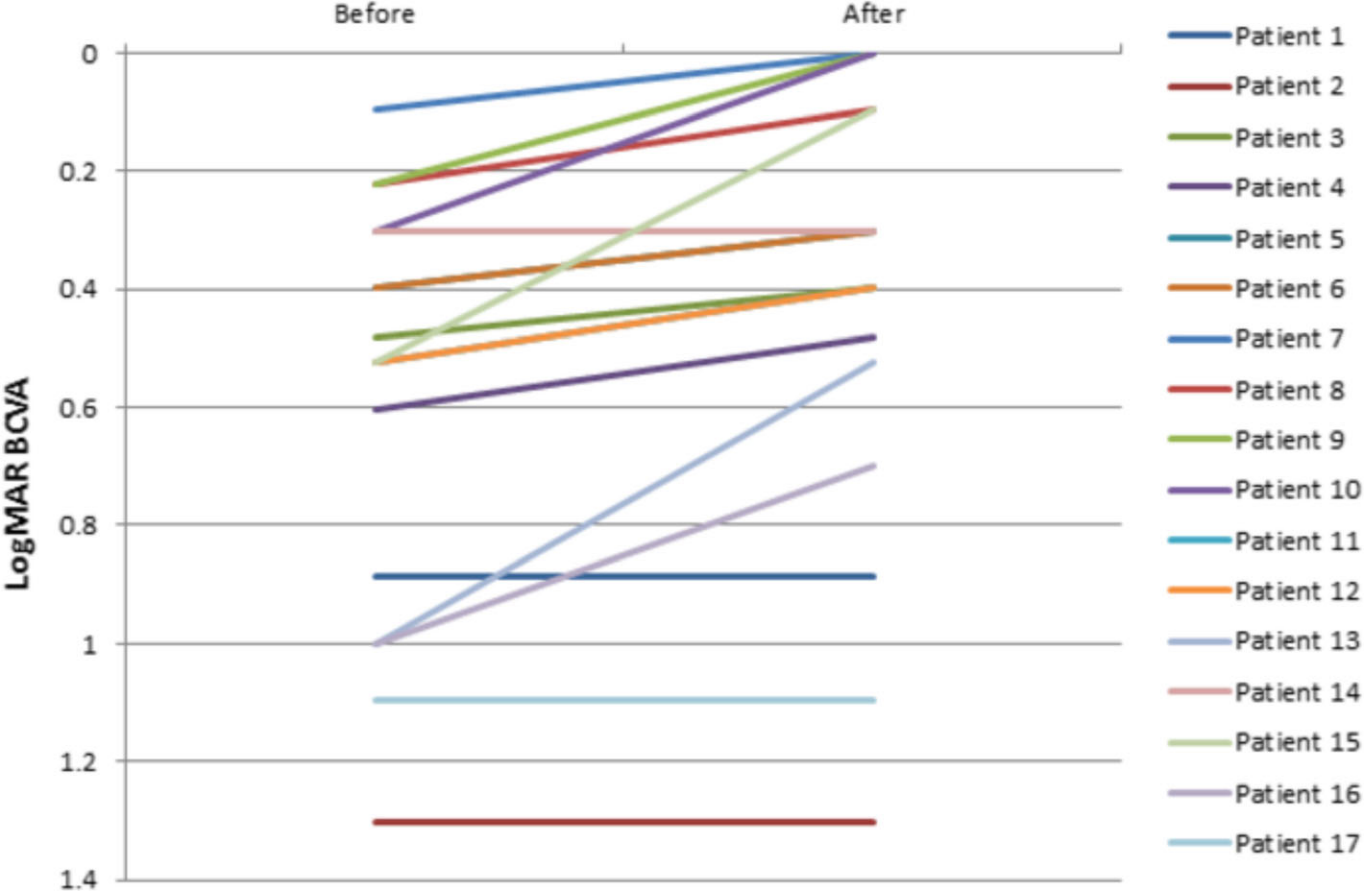
Change in best corrected visual acuity (BCVA) with the treatment for each patient evaluated

**Fig. 4:**
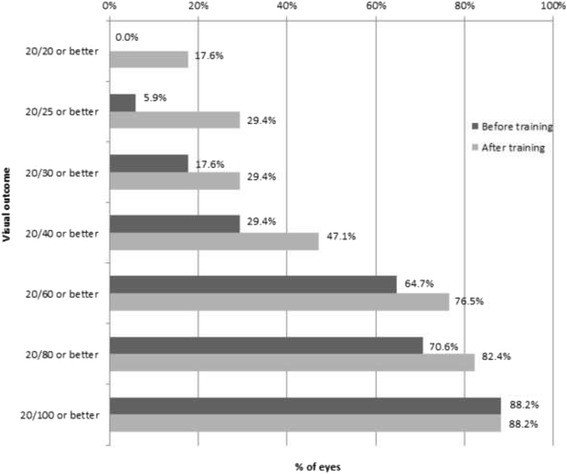
Distribution of best corrected visual acuity data in the analyzed sample before and after visual traning

Table 1 summarizes the main clinical data of patients included in the study. Mean spherical equivalent was +0.09 ± 2.13 and +2.01 ± 4.07 D in the healthy and amblyopic eye, respectively (*p* = 0.004, Student t test). Likewise, Table 1 includes BCVA of the amblyopic eye and stereoacuity data before and after the 8 dichoptic training sessions. As shown, BCVA improved significantly from a mean logMAR BCVA value of 0.58 ± 0.35 before training to a mean post-training value of 0.43 ± 0.38 (*p* < 0.01, Student t test) (Fig. 3). Snellen BCVA of the amblyopic eye ranged before training from 20/400 to 20/25 and from 20/400 to 20/20 after training (Fig. 3). A total of 29.41% and 47.06% of eyes achieved a BCVA of 20/40 or better before and after training, respectively (Fig. 4). Most of the patients gained lines (1.5 logMAR line on average) of BCVA except those three with the lowest BCVA (1.30, 1.10 and 0.9 logMAR) and one patient with 0.30 logMAR BCVA (Fig. 4). In these four cases, no change in BCVA was observed.

**Table 1:** Baseline characteristics and results of the sample of patients that performed the virtual reality dichoptic training using the Oculus Rift virtual reality head mounted display

Patient	Healthy eye (Dsph. Dcyl)		Amblyopic eye (Dsph. Dcyl)		BCVA -before DT	BCVA -after DT	Stereoacuity -before DT	Stereoacuity -after DT
1	+0.5	+1	−0.5	+3.50	0.9	0.9	nil	nil
2	+0.75	+1.5	+2.37	+0.75	1.3	1.3	nil	400
3	+0.25	+0.5	+4.5	+2.5	0.5	0.4	nil	140
4	−2.5	+1	−3.5	+1	0.6	0.5	nil	50
5	+0.50	0	+7.00	+0.5	0.4	0.3	400	400
6	+2	+0.5	+3.00	+1.0	0.4	0.3	200	200
7	−0.12	+0.75	+1.5	+1.0	0.1	0.0	70	50
8	+0.25	+0.5	+3.00	+0.5	0.2	0.1	140	20
9	+0.75	0	+3.75	+0.5	0.2	0.0	400	160
10	+0.25	+0.5	+1.00	+1.5	0.3	0.0	200	20
11	+0.5	+0.5	+4.25	+1.0	0.5	0.4	400	140
12	+0.50	0	+2.25	+1.5	0.5	0.4	nil	400
13	+0.25	0	+1.75	+3.0	1.0	0.5	niI	400
14	+0.5	+1.0	+2.50	0	0.3	0.3	400	50
15	+0.5	+0.5	+4.0	+1.0	0.5	0.1	160	20
16	−8.0	+1.0	−12.0	+2	1.0	0.7	nil	nil
17	0	0	+1.87	+0.75	1.1	1.1	nil	200

*Abbreviations*: *SE* sperical equivalent, *BCVA* best corrected visual acuity, *DT* dichoptic training

**Fig. 5:**
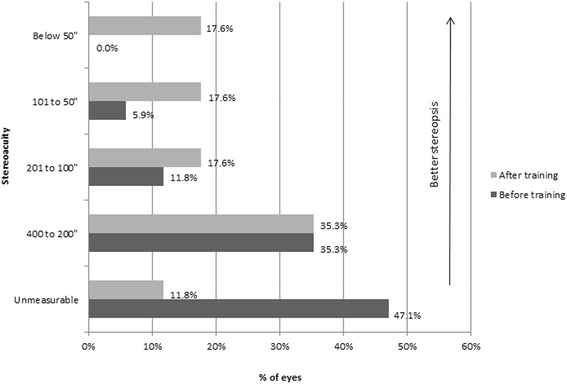
Change in stereoacuity with the treatment for each patient evaluated

**Fig. 6:**
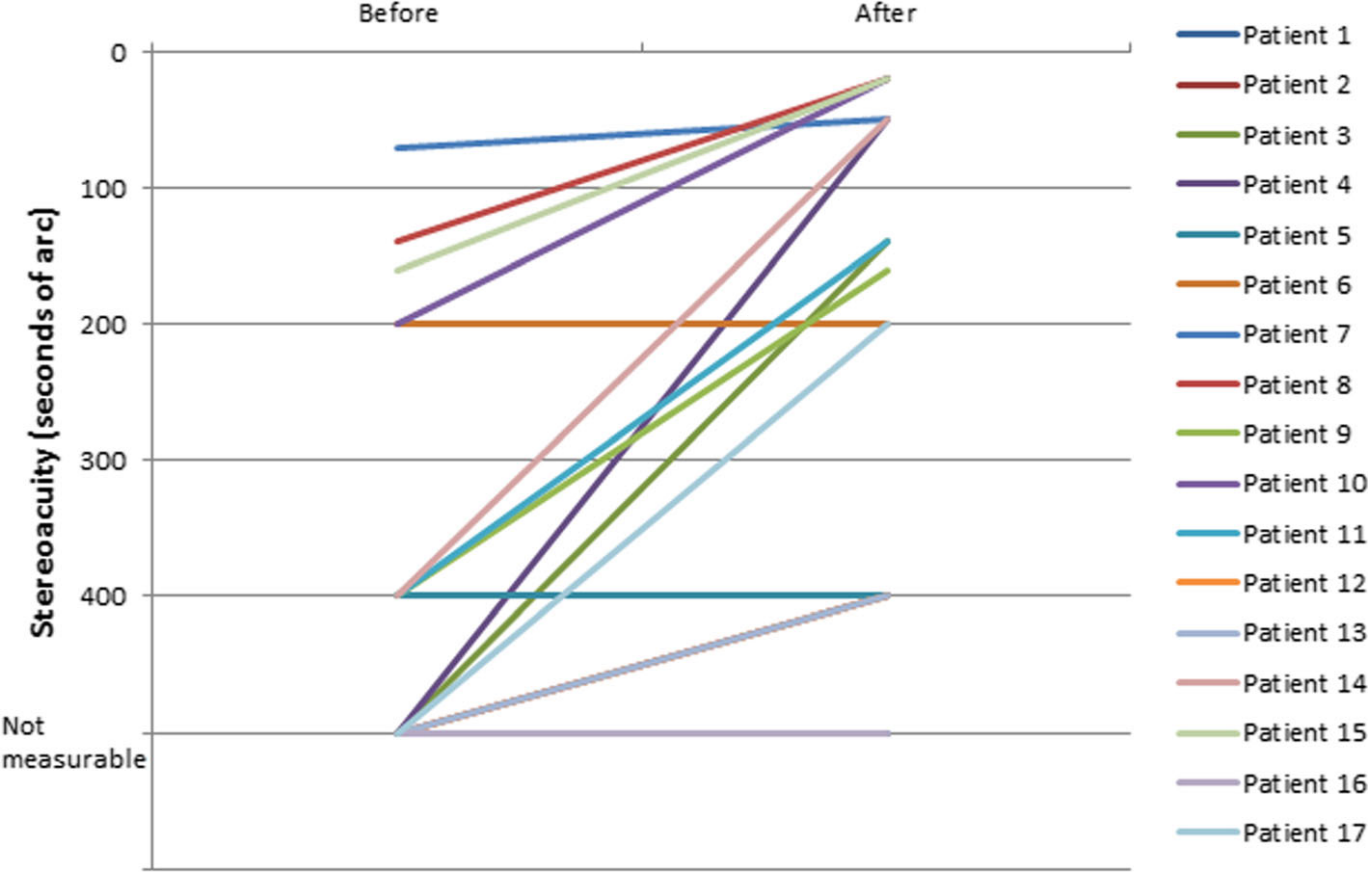
Distribution of stereoacuity data in the analyzed sample before and after visual traning

Stereoacuity was measured using the Stereo Randot graded circle test, with the ability of measuring stereoacuities from 400 to 20 s of arc. Mean stereoacuity changed from a value of 263.3 ± 135.1 before dichoptic training to a value of 176.7 ± 152.4 s of arc after training (Table 1) (Fig. 5). This change was statistically significant (*p* < 0.01, Wilcoxon signed-rank Test). A total of 8 patients (47.1%) before dichoptic treatment had unmeasurable stereoacuity with the method used while this only occurred in 2 patients (11.8%) after training (Fig. 6).

Subjectively, patients did not refer problems after dichoptic training. Occasionally some patients felt tired after training and reported a sensation of pulling behind the amblyopic eye.

## Discussion

In young children with anisometropic amblyopia, a period of 16–22 weeks of treatment with optical correction alone is enough in the majority of cases to overcome the suppression and leading to an improvement of visual acuity to 2 lines or more [13]. In nearly one-third of amblyopic children, the treatment with optical correction is enough to completely resolve the amblyopia [13]. For older children, patching or atropine therapy is a complementary effective treatment for amblyopia. After a stable visual acuity is achieved with spectacle wear, older children require 120 h of treatment to achieve 1 line improvement [14]. However, binocular vision plays an important role in amblyopia and this brings new approaches to the development of more effective treatments [15]. Recent studies show that binocular-dichoptic training may result in significantly greater learning effects than monocular training [11, 12, 16]. Li and colleagues [16] demonstrated that 9 h of training with dichoptic movies over period of 2 weeks resulted in 1 to 4 lines of visual improvement in children (4–10 years).

In adults, the scientific evidence of the effectiveness of the treatment of amblyopia is scarce [17, 18]. In the current study, we report the results of a preliminary study evaluating the outcomes of dichoptic visual training using a virtual reality head mounted display in adults with anisometropic amblyopia. An improvement in BCVA of 1.5 logMAR lines on average has been found in our series. These results are consistent with those reported also in previous study evaluating the effects of other dichoptic training methods [11, 19]. A recent study demonstrated that just 10 h of dichoptic videogame play improved visual acuity by more than 0.16 LogMAR [11]. An increase of 0.34 logMAR in BCVA has been reported by Spiegel and colleagues [20] after 10 to 65 min of training with a Tetris dichoptic video game. In contrast, monocular video game play with patching of fellow eye has shown to improve visual acuity in adults with amblyopia by an average of 1.6 lines LogMAR after 40 h of training [12]. The use of dichoptic training may be then more effective than monocular training in amblyopia but this is something that should be investigated further in controlled comparative studies. In our series, BCVA did not improve in three patients. This is consistent with previous studies showing that a percentage of amblyopic patients ranging from 10 to 50% may fail to achieve normal visual acuity even after extended periods of treatment [14]. Specifically, our younger patients showed an increase in BCVA, suggesting that lower brain plasticity might be one of the possible reasons for the absence of BCVA improvement in three cases [21]. Another reason might be the decreased function of afferent innervation in either grey or white central nervous system matter as shown in previous magnetic resonance studies [22]. Likewise, motivation of patients, as with other forms of perceptual learning, may play a significant role on the outcomes. Future studies are needed to determine the real causes for the absence of improvement with dichoptic treatment in some amblyopic patients.

In our sample, stereoacuity improved in 7 out of 10 patients, whereas in other studies evaluating other modalities of binocular treatment have reported improvement rates of 50% to 60% [23–27]. Possibly, the use of virtual reality may play a role in this enhanced stereoacuity after dichoptic training in Oculus Rift. Fulvio and collegaues [28] demonstrated that Head tracking in virtual reality displays reduces the misperception of 3D motion. More research on this issue is needed in order to evaluate the potential usefulness of virtual reality training for the improvement of stereopsis in amblyopia.

The preliminary results of our study indicate the potential for a new treatment for adulthood amblyopia. It is still necessary to perform a controlled clinical trial evaluating this potential treatment option for amblyopia, not only in adults, but also in children. Our results suggests that suppression of the amblyopic eye gates plasticity within the adult amblyopic visual cortex and therefore some degree of residual cortical plasticity can be unmasked in the adult brain [29]. It should be also considered that motivational effects associated with video game plays may play also an important role in neuronal plasticity of the central nervous system.

In the current preliminary study, we have used a protocol of treatment of 8 sessions of treatment during 1 month (2 sessions/weekly). The reason for the selection of this protocol was based on the consideration that the compliance may be better if the treatment was shorter and also on previous experiences demonstrating that the greatest improvement with perceptual training is achieved in the first eight sessions of treatment [21, 30]. Future studies must be conducted to investigate which is the best protocol of treatment using dichoptic training combined with virtual reality.

This preliminary study has some limitations, including the sample size, the short follow-up and the absence of a control group. Although we do not have complete data yet on long term stability of BCVA after the treatment, we are planning to check the patients treated in the current study at 6 and 12 months after finishing the treatment. Three patients have been already checked up at 6 months after finishing the treatment and all of them had a stereo-acuity of 100 s of arc or better and stable BCVA. In addition, the inclusion of a placebo or control group would have been recommendable. However, the aim of this preliminary study was to show the potential viability of the treatment and to confirm if future controlled and randomized studies were worthy to be done. Finally, we have used a stereopsis test that can only measure values of 400 s of arc as maximum. A better choice would have been to use a stereopsis test allowing measuring stereopsis in a wider range. This will be considered in future studies.

## Conclusions

In conclusion, this preliminary study shows the potential usefulness of dichoptic training using a virtual reality head mounted display in the treatment of adult anisometropic amblyopia. Future clinical trials are needed to confirm this preliminary evidence as well as studies evaluating the potential benefit in stereopsis outcome of using a virtual reality head mounted display.

## Abbreviations

3D: Three dimensions
BCVA: Best corrected visual acuity
LCD: liquid crystal display
